# Midgut microbiota affects the intestinal barrier by producing short-chain fatty acids in *Apostichopus japonicus*

**DOI:** 10.3389/fmicb.2023.1263731

**Published:** 2023-10-17

**Authors:** Mingshan Song, Zhen Zhang, Yanan Li, Yangxi Xiang, Chenghua Li

**Affiliations:** ^1^State Key Laboratory for Managing Biotic and Chemical Threats to the Quality and Safety of Agro-products, Ningbo University, Ningbo, China; ^2^Laboratory for Marine Fisheries Science and Food Production Processes, Qingdao National Laboratory for Marine Science and Technology, Qingdao, China

**Keywords:** short-chain fatty acids, intestinal permeability, acetic acid, *Vibrio splendidus*, *Apostichopus japonicus*

## Abstract

**Introduction:**

The intestinal microbiota participates in host physiology and pathology through metabolites, in which short-chain fatty acids (SCFAs) are considered principal products and have extensive influence on intestine homeostasis. It has been reported that skin ulceration syndrome (SUS), the disease of *Apostichopus japonicus* caused by *Vibrio splendidus*, is associated with the alteration of the intestinal microbiota composition.

**Method:**

To investigate whether the intestinal microbiota affects *A. japonicus* health via SCFAs, in this study, we focus on the SCFA profiling and intestinal barrier function in *A. japonicus* treated with *V. splendidus*.

**Results and discussion:**

We found that *V. splendidus* could destroy the mid-intestine integrity and downregulate the expression of tight junction proteins ZO-1 and occludin in *A. japonicus*, which further dramatically decreased microorganism abundance and altered SCFAs contents. Specifically, acetic acid is associated with the largest number of microorganisms and has a significant correlation with occludin and ZO-1 among the seven SCFAs. Furthermore, our findings showed that acetic acid could maintain the intestinal barrier function by increasing the expression of tight junction proteins and rearranging the tight junction structure by regulating F-actin in mid-intestine epithelial cells. Thus, our results provide insights into the effects of the gut microbiome and SCFAs on intestine barrier homeostasis and provide essential knowledge for intervening in SUS by targeting metabolites or the gut microbiota.

## 1. Introduction

The intestinal microbiota is a wild and diverse microbial ecosystem that has co-evolved with host species (Spiljar et al., 2017). In recent years, several studies have concentrated on the interaction between the host and the intestinal microbiota (Bonder et al., 2016; Wang et al., 2016; Rothschild et al., 2018). The microbial community was found to be affected by host genetic variation; for example, pathological shifts of the microbial community were found to be induced by host genetic susceptibilities in inflammatory bowel disease (IBD) (Plichta et al., 2019). Furthermore, the microbiota and metabolites derived from the microbiota have adverse effects on the immune system and physiology of the host in IBD (Qin et al., 2018; Plichta et al., 2019). It has been demonstrated that the gut microbiome directly participates in the pathogenesis of various pathological activities, such as circulatory disease, obesity, IBD, and autism (Marchesi et al., 2007; Turnbaugh et al., 2007; Finegold, 2008; Holmes et al., 2008). Therefore, the intestinal microbiota performs various essential biochemical functions for the host, and disorders of the microbiome are associated with several human diseases. Given the interaction between the intestinal microbiota and host health in mammals by regulating physiological activities (Turnbaugh et al., 2006), immune response (Mazmanian et al., 2005), energy metabolism, and the response of intestinal homeostasis to epithelial cell injury (Rakoff-Nahoum et al., 2004), the intestinal microbiota is increasingly being valued for its crucial role in the maintenance of aquatic invertebrate health. Obvious correlations between the intestinal microbiota and physiological activities, such as neurotransmission, detoxification, oxidative stress, and intestinal epithelium integrity, were observed in aquatic invertebrates as well (Falcinelli et al., 2015). Recent studies have demonstrated that the gut microbiome of grass carp plays a role in carbohydrate turnover and fermentation, and the intestinal microbiota of *Nereis succinea* plays a role in biodegrading various organic pollutants (Wu et al., 2015; Wang et al., 2020). Moreover, gut microbiome disorders are associated with diverse aquatic animal disease processes. Enteritis in carps has been found to change the specific metabolic pathway, accompanied by a significant increase in members of the genera *Pseudomonas, Flavobacterium, Caulobacter, Rhodobacter, Planctomyces, Methylocaldum*, and *Dechloromona* (Tran et al., 2018). Hong et al. (2020) found that intestinal beneficial bacteria decreased and pathogens increased in Chinese mitten crabs exposed to imidacloprid. Zhang et al. (2018) demonstrated that *A. japonicus* afflicted with skin ulceration syndrome (SUS) had higher Firmicutes abundance and lower Verrucomicrobia abundance, while *Lactococcus garvieae* exhibited considerable abundance in sea cucumbers afflicted with SUS.

The sea cucumber *Apostichopus japonicus* (Skelenka) is an economically and ecologically important cultivation in coastal ecosystems of the western North Pacific Ocean (Zhang et al., 2018), which is known as the “ecosystem engineer” because it facilitates nutrient exchange and the carbonate cycle in the water–sediment interface (Schneider et al., 2011). However, SUS, a disease mainly caused by *Vibrio splendidus*, has led to huge economic losses and has been considered the main disease in *A. japonicus* aquaculture (Deng et al., 2008). Because SUS can induce intestinal microbiota dysbiosis, it is important to investigate the interaction between SUS and intestinal microbiota disorder. Then, we can develop a control strategy for SUS by taking advantage of the susceptibility of intestinal microorganisms and targeting them.

The digestive tract tissue of *A. japonicus* is composed of epithelial tissue, inner connective tissue, muscle layer, outer connective tissue, and peritoneum (Liao, 1997). Intestinal epithelium is the first protector of the host body against pathogenic microorganisms, and it is the structural basis for maintaining intestinal selective permeability and barrier function (Turner, 2006; Groschwitz and Hogan, 2009; Liang and Weber, 2014; Zhang et al., 2015). Therefore, the intestinal epithelium plays a significant role in preserving the stability of the host and external environment. Cell junctions in the intestinal epithelial tissue, such as the tight junction (TJ), gap junction (GJ), adhesion junction (AJ), and desmosome, jointly form the intestinal mechanical barrier. The tight junction is mainly composed of zonula occludens (ZO-1, ZO-2, and ZO-3), occludin, claudin, among other, and they play a vital role in maintaining the stability of the intestinal barrier structure and function (Haskins et al., 1998). Vaziri et al. (2013) found that the intestinal microbiota of chronic kidney disease patients changes rapidly and leads to damage, characterized by increased intestinal permeability in the intestinal barrier. Many studies have demonstrated that intestinal permeability significantly increases due to the downregulation of occludin and ZO-1 expression in IBD patients (Piche et al., 2008; Bertiaux-Vandaële et al., 2011). Furthermore, *Escherichia coli* and *Salmonella typhimurium* can disrupt the integrity of the intestinal barrier by altering TJ proteins' expression and arrangement (Berkes et al., 2003; Chin et al., 2006). The increase in probiotics *Lactobacillus sakei* OK67 abundance can reduce inflammation response and increase the ZO-1 expression (Lim et al., 2016).

Based on intestinal microecology, previous studies have continuously explored the effect of intestinal microbiota dysbiosis on the intestinal barrier. Therefore, it is important to search for substances that play an essential role in modulating the effect of intestinal microbiota on the intestinal barrier. The metabolites produced by the gut microbiota exert their effects on the host as signaling molecules and substrates for metabolic reactions. Short-chain fatty acids (SCFAs) could participate in host health as the most abundant microbiome-produced molecules that possess fewer than six carbons, including acetate, propionate, butyrate, pentanoate, and caproate. It has been reported that SCFAs are related to the maintenance of the microbial ecosystem (Duncan et al., 2007), and the profile variation of SCFAs can reflect the metabolic interaction between microorganisms. Some studies have discussed the effect of SCFAs on the intestinal barrier *in vivo* and *in vitro* (Miyoshi et al., 2008). SCFAs promote the recovery of the intestinal epithelium and induce a reversible reduction in paracellular permeability in a concentration-dependent manner *in vitro* (Wilson and Gibson, 1997; Mariadason et al., 1999). Butyrate promotes the intestinal barrier by modulating the combination of TJ in Caco-2 cell monolayers (Peng et al., 2009). Mátis et al. (2022) demonstrated that butyrate has positive modulatory effects on tight junction proteins, namely, claudin-1 and claudin-3, by oral butyrate supplementation in the chicken intestine.

The *A. japonicus* alimentary canal includes the foregut, midgut, and hindgut (Zamora and Jeffs, 2011). The foregut is used to transport food, the midgut is used to digest food and absorb nutrition, and the hindgut is used for excretion. The microbial diversity of the midgut and hindgut is greater than that of the foregut in matters of species and quantity (Gao et al., 2014; Wang et al., 2018). Compared with the hindgut, the midgut can better absorb and utilize SCFAs due to its striated border and villous epithelium. Furthermore, Dong et al. (2009) stated that pathogens could directly interact with and adversely affect the host by destroying the physical barrier of the midgut. Therefore, we regarded the midgut as the object to explore the interaction between the intestinal microbiota and *A. japonicus* mediated by SCFAs. The invasion of *V. splendidus* significantly altered the intestinal microbiota abundance and species, accompanied by a change in metabolomic profiles (Zhang et al., 2018). Therefore, we hypothesized that SCFA profiles are altered during the occurrence of SUS, that the intestinal microbiota can interact with *A. japonicus* via SCFAs, and that SCFAs can alleviate the effect of *V. splendidus* on the intestinal barrier.

To address the hypothesis above, in the present study, a comprehensive exploration of the variation in the content of SCFAs in *A. japonicus* was achieved by combining the intestinal microbiota aberrations under *V. splendidus* infection. The protective effect of SCFAs on the intestinal barrier was first validated by investigating the tight junction expression and rearrangement. These valuable findings could provide a new prevention and control strategy for SUS.

## 2. Materials and methods

### 2.1. Infection experiment

*A. japonicus* with an average weight of 200 ± 15 g was collected from a farm in Dalian (Liaoning Province, China). The collected *A. japonicus* was acclimatized for 1 week before the experiment in a clear pool, and it was fed with a mixture of feed. One-third of the water was exchanged every day. *Vibrio splendidus* was grown in 2216E media at 28°C to OD_600_ = 1.0 for the bacterial suspension. The supernatant of the bacterial suspension was removed by centrifuging at 6,000 × g for 10 min. The bacterial precipitation was suspended in seawater and used to infect the sample *A. japonicus* with appropriate concentrations (1 × 10^7^ CFU ml^−1^) (Zhang et al., 2018). Fifty sea cucumbers were infected with 1 × 10^7^ CFU ml^−1^ of *V. splendidus* in a tank containing 1,000 L seawater. We collected eight samples at 0, 12, 48, and 96 h after *V. splendidus* infection and SUS. The midgut tissues were collected, and the contents were sampled for the following experiments.

### 2.2. Histopathological and immunofluorescence analyses

Abdominal incisions were performed on three *A. japonicus* in each group of 0, 12, 48, 96 h, and SUS to collect midgut tissues, and then, the midgut was cutoff using small surgical scissors. The midgut tissues were fixed with paraformaldehyde and embedded with paraffin. The slices (5 μm-thick) were stained with hematoxylin and eosin (H & E) and analyzed under a light microscope (Carl Zeiss, German). For the midgut tissue immunofluorescence analysis, the midgut tissues of three *A. japonicus* in each group of 0, 12, 48, 96 h, and SUS were embedded in an OCT compound (optimal cutting temperature compound), frozen with liquid nitrogen, and stored in −80C. Slices of 10 μm were cut, fixed with paraformaldehyde for 20 min, penetrated with PBS containing 0.5% Triton X-100 for 20 min, blocked for 30 min in an antibody diluent, and stained with the primary antibodies ZO-1 and occludin (1:200, Beyotime Biotechnology, China) for 30 min in the antibody diluent. After three washes, the sections were stained with secondary antibodies Alexa Fluor 555-labeled Goat-anti-Rabbit IgG (1:1,000, Biotechnology, China) and Alexa Fluor 488-labeled Goat-anti-Rabbit IgG (1:1,000, Biotechnology, China) for 30 min. After three washes, the nucleus was stained using DAPI (1:10,000, Beyotime Biotechnology, China) for 5 min. The slices were mounted, and the images were analyzed under a Zeiss LSM 880 confocal laser scanning microscope (Carl Zeiss, German).

### 2.3. Midgut microbiota analysis

Gut content samples were collected from eight *A. japonicus* infected with *V. splendidus* at 0, 12, 48, 96 h, and SUS, and genomic DNA extraction was performed. The primer pairs 338F (5′-GTACTCCTACGGGAGGCAGCAG-3′) and 806R (5′-GGACTACHVGGGTWTCTAAT-3′) were used to amplify the V3-V4 region of the 16S rRNA gene (Zhang et al., 2018). The 16S rRNA sequence was assembled and filtered to obtain valid data (clean data). QIIME2 was used to control the quality of the clean data. The sequences were clustered and classified into operational taxonomic units (OTUs) at a 97% threshold in DADA2 in the 0, 12, 48, 96 h, and SUS groups, wherein the feature sequence was annotated by employing the blast method according to the SILVA database (Quast et al., 2013; Abellan-Schneyder et al., 2021). Bacterial classification and analysis were performed by referring to our previous study (Zhang et al., 2018). Spearman's correlation analysis was conducted to analyze the correlations between the key taxa and the SCFAs using R (version 4.1.0) (Livak and Schmittgen, 2001; Fagan et al., 2007).

### 2.4. Midgut content collection and targeted SCFAs profiling

Eight *A. japonicus* were taken from each group of 0, 12, 48, and 96 h after *V. splendidus* infection and SUS. The abdominal anatomy of the *A. japonicus* was immediately examined after sterilizing the body surface with 75% alcohol. The entire intestine was taken from the body cavity of the sea cucumbers, and the esophagus, stomach, and hindgut were cut off in a sterile environment. Subsequently, the contents of the midgut were squeezed into a 2-mL sterile tube and immediately immersed in liquid nitrogen. The SCFA concentration in the midgut contents was measured by gas chromatography/mass spectrometry (GC/MS). For the SCFA assay, 50 mg gut content was homogenized for 1 min with 50 μl 15% phosphoric acid, 100 μl 125 μg/ml isohexanoic acid solution, and 400 μl ether; then, it was centrifuged at 12,000 rpm at 4°C for 10 min. The supernatants were used for GC/MC (TRACE 1310-ISQ LT, Thermo, USA) analysis. Ether was used to prepare pure standards of acetic acid (CAS 64-19-7), propionic acid (CAS 79-09-4), butyric acid (CAS 107-92-6), isobutyric acid (CAS 79-31-2), valeric acid (CAS 109-52-4), isovaleric acid (CAS 503-74-2), and caproic acid (CAS 142-62-1) in ten mixed standard concentration gradients: 0.1, 100, 10, 0.02, 250, 25, 2, 0.5, 500, and 50 μg/ml. A chromatographic column Agilent HP-INNOWAX capillary column (30 m × 0.25 mm ID × 0.25 μm) was used to separate the metabolites. Helium was passed through the column as a carrier gas at a constant flow rate of 1 ml/min. The initial temperature was 90°C, which was ramped to 120°C at a rate of 10°C/min, 150°C at a rate of 5°C/min, 250°C at a rate of 25°C/min, and finally kept at 250°C for 2 min. The linearity was investigated by taking the concentration of the standard as the abscissa and the peak area ratio of the standard to the internal standard as the ordinate. The obtained linear regression equation of each substance is shown in Supplementary Table 1. The correlation coefficient was *r* > 0.99.


$$\begin{array}{l} \text{SCFAsconcentration}\left(\text{μg}/\text{ml}\right)=\left(\text{peakarea ratio}-\text{b}\right)/\text{a} \end{array}$$

SCFAs content (μg/g)= concentration (μg/ml) × volume (ml)/sampling amount (mg) × 1,000

^*^(The values of a and b are shown in Supplementary Table 1).

The SCFA data were analyzed using SIMCA-P software.

### 2.5. RNA analysis

The midgut tissues of four individuals were sampled as described above, and the RNA was extracted using TRIzol reagent (Takara, Otsu, Japan). The RNA was reversely transcribed into cDNA using the Reverse Transcription Kit (Takara, Otsu, Japan) by following the manufacturer's protocol. qRT-PCR was used to analyze the occludin and ZO-1 expression, and it was executed on the Applied Biosystems 7500 real-time PCR system (Supplementary Table 2). The total volume of the reaction was 20 μl, containing 10 μl of TB GREEN^TM^ Premix Ex Taq II, 8 μl of cDNA, 0.8 μl each of forward and reverse primers, and 0.4 μl of ROX (Takara, Otsu, Japan). The reaction process consisted of the following steps: 5 min of initial denaturation at 95C, 15 s of denaturation at 95C, and 30 s of annealing and extension at 60C, comprising 45 cycles in total. The result was represented by 2^−Δ*ΔCt*^, and the data were normalized by using β-actin as a reference gene (Evans and Surprenant, 1992).

### 2.6. Western blotting and ELISA

Hundred milligrams of midgut tissues from three individuals were washed with cold PBS, and protein was extracted from them using RIPA lysis (Sangon, China); the protein concentration was quantified using the BCA assay kit. Equal amounts of protein (50 μg) were separated by sodium dodecyl sulfate-polyacrylamide gel electrophoresis (SDS-PAGE) and then transferred to nitrocellulose filter (NC) membranes. After blocking the NC membranes with 5% non-fat milk in TBS at room temperature for 1 h, the membranes were incubated with the following primary antibodies at 4°C overnight: ZO-1 (1:2,000, Beyotime Biotechnology, China), occludin (1:2,000, Beyotime Biotechnology, China), and β-actin (1:5,000, Proteintech Group, USA). The membranes were incubated with HRP-conjugated Goat-anti-Rabbit IgG at room temperature for 1 h after washing three times with TBST (Beyotime Biotechnology, China). The immunoreactive proteins were analyzed using the Omega Lum C imaging system (Aplegen, California, USA). The band intensity was quantified using the Image J software.

The midgut tissues were collected, and 1 mL PBS homogenate was added. The supernate was kept after centrifugation for 10 min at 3,000 rpm. The ZO-1 and occludin were measured in the supernate following standard protocols, with three replicates in each group. The rat tight junction protein 1 (ZO-1) ELISA Kit (KT, China) and the Rat occludin ELISA Kit (KT, China) were used for the detection of ZO-1 and occludin.

### 2.7. Acetate treatment

*A. japonicus* with an average weight of 200 ± 15 g was acquired from a farm in Dalian (Liaoning Province, China). The collected *A. japonicus* was acclimatized for 1 week before the experiment in a clear pool, and it was fed with a mixture of feed. One-third of the water was exchanged every day. The sample *A. japonicus* was randomly assigned to four groups (four sea cucumbers per group): the control group (Control), the acetate feeding group (Acetate), the *V. splendidus* infection group (*V. splendidus*), and the acetate feeding + *V. splendidus* infection group (Acetate + *V. splendidus*). *A. japonicus* in the acetate and acetate + *V. splendidus* groups was orally administered with 500 mg/kg acetate in their normal feed, while *A. japonicus* in the control and *V. splendidus* groups was orally administered normal feed made with sea mud powder, *Sargassum thunbergii* powder, and *Spirulina* powder. After 8 weeks, midgut tissues were collected from each *A. japonicus* for histopathological analysis, immunofluorescence staining, qRT-PCR, and Western blotting for examining the occludin and ZO-1 expression, as described in Section 2.2.

### 2.8. Cell culture

To culture the primary midgut epithelial cells of *A. japonicus*, the body surface of three *A. japonicus* individuals was sterilized with 75% ethanol. Then, the midgut tissues of the three individuals were collected; the mesentery was removed, and the lumen of the midgut segments was flushed thrice with D-Hanks, which contained 200 U/ml penicillin and 200 μg/ml streptomycin sulfate. The midgut segments were turned over so that the intestinal mucosa would face outward. After washing the intestinal mucosa with the D-Hanks cleaning solution, it was placed in a culture dish, and scraped with a sterile scalpel; the cleaning solution was used to repeatedly clean the scraped epithelial cells (Peerapen and Thongboonkerd, 2021). The cells were seeded on 12-well microplates and grown in Leibovitz's L-15 cell medium (Invitrogen, USA) containing 100 U/ml penicillin and 100 μg/ml streptomycin sulfate at 16°C. Three wells were first treated with one treatment and later treated with acetate (0.07 g/ml) and LPS (10 μg/ml) for 24 h.

For the immunofluorescence analysis of intestinal epithelial cells, the cells were fixed with paraformaldehyde and incubated with ZO-1 antibody (1:200, Beyotime Biotechnology, China) and Coralite^®^488-conjugated Goat Anti-Rabbit IgG (1:200, Proteintech Group, USA). The nucleus was stained using DAPI (1:10,000, Beyotime Biotechnology, China) for 5 min. Analysis was performed under a Zeiss confocal microscope (Carl Zeiss, LSM880, Germany) and using the Image J software by calculating cell fluoroscence.

### 2.9. Statistical analysis

SPSS V23.0 statistical software (SPSS Co., Ltd., Chicago, Illinois, USA) was used for statistical analysis. All data were expressed as mean ± standard deviation. If there were more than two groups, a two-sided unpaired *t*-test was performed for analysis. The differences among the three groups were analyzed by one-way analysis of variance (ANOVA) with Duncan's range tests. Pearson correlation analysis was also performed. The difference was considered to be within a *p*-value of <0.05.

## 3. Results

### 3.1. *Vibrio splendidus* infection modulates permeability of midgut in *A. japonicus*

**Figure 1:**
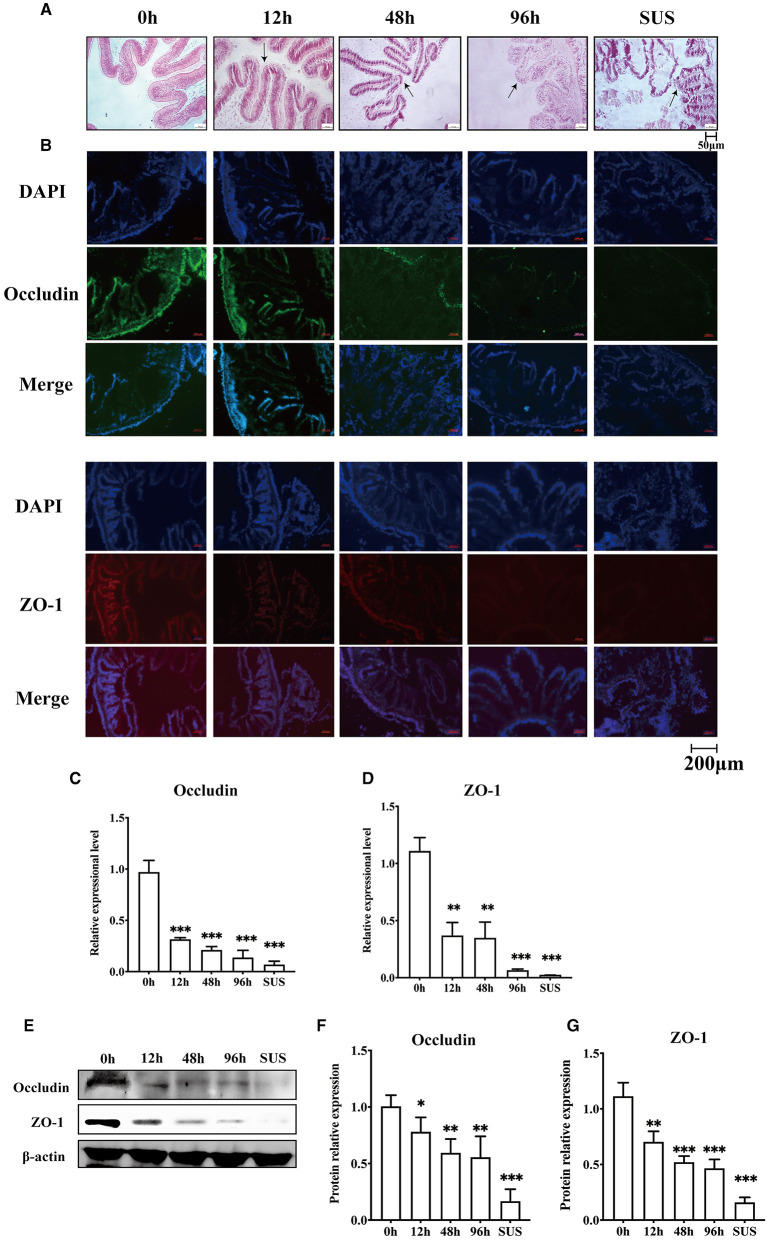
Changes in the intestinal mechanical barrier of *A. japonicus* with *V. splendidus* infection. **(A)** H&E staining of the section of the *A. japonicus* intestine. **(B)** Immunofluorescence of ZO-1 and occludin in the *A. japonicus* intestine. **(C, D)** RT-PCR analysis of occludin and ZO-1 transcript levels from the intestine tissue. **(E–G)** The western bloting was used to determine the expression level occludin and ZO-1 with β-actin as the reference in the intestine tissue. Asterisks indicate significant differences: *p < 0.05, **p < 0.01, and ***p < 0.001.

To confirm whether *V. splendidus* can influence the intestine barrier, we analyzed the variation in the midgut morphology and the disruption of the intestinal tight junction before and after *V. splendidus* infection. The midgut morphology results showed that the intestinal striated border gradually fell off, and the morphological structure of the epithelial tissue was disordered. The absorbing cells became necrotic, they shed, and the cell arrangement gradually loosened as the infection progressed over time (Figure 1A). There was obvious shedding of the intestine villi and striated borders in SUS *A. japonicus*. To determine if *V. splendidus* mediated intestinal barriers by targeting ZO-1 and occludin, their protein expressions were assayed. The result indicated that the fluorescence signal of ZO-1 and occludin gradually disappeared during *V. splendidus* infection. Consistently, the occludin and ZO-1 mRNA levels were significantly decreased; they reached the lowest level in SUS *A. japonicus* and only 0.02- and 0.07-fold, respectively, in the groups treated for 0 h (Figures 1C, D). Furthermore, the Western blotting results confirmed that occludin and ZO-1 downregulation was induced by *V. splendidus* (Figures 1E–G), suggesting that *V. splendidus* caused damage to the intestinal barrier by downregulating tight junction proteins.

### 3.2. *Vibrio splendidus* infection altered midgut microbiota profiles

**Figure 2:**
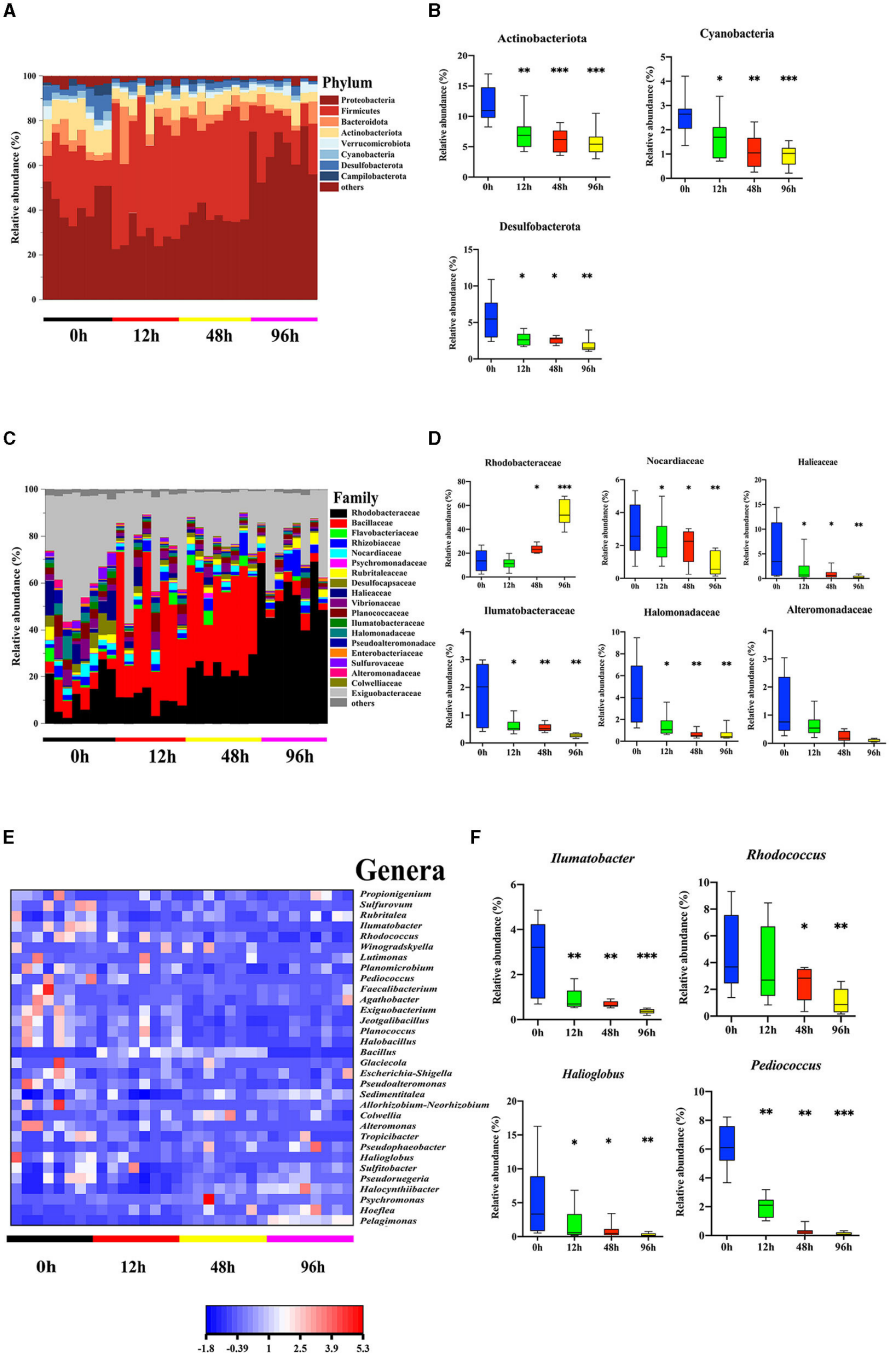
*Vibrio splendidus* infection modulated the midgut microbiota in the sea cucumber. **(A, B)** Microbial abundance and significant differences in the bacterial taxonomy at the phylum level. **(C, D)** Microbial abundance and significant differences in the bacterial taxonomy at the family level. **(E, F)** Relative abundance of the genera heatmap and significant differences in the bacterial taxonomy at the genus level. Asterisks indicate significant differences: *p < 0.05, **p < 0.01, and ***p < 0.001.

Our previous study revealed differences in the microbiota community in the mid-intestine between healthy and diseased *A. japonicus* (Zhang et al., 2018). To characterize the alteration of the midgut microbiota during the *V. splendidus* infection, the 16S rRNA gene sequence data of *A. japonicas*, which were treated with *V. splendidus* for 0, 12, 48, and 96 h, were further analyzed. Seven predominant phyla (*Proteobacteria, Firmicutes, Bacteroidota, Verrucomicrobiota, Cyanobacteria, Desulfobacterota*, and *Campilobacterota*) were detected, and all of them displayed significant alterations in abundance after *V. splendidus* treatment (Figure 2A; Supplementary Table 3). Taxonomic analysis of the phylum distribution showed significant reductions in Actinobacteriota, Cyanobacteria, and Desulfobacterota after *V. splendidus* treatment (Figure 2B). At the family level, Rhodobacteraceae increased in abundance, while Nocardiaceae, Halieaceae, Ilumatobacteraceae, Halomonadaceae, and Alteromonadaceae were obviously decreased in abundance in the infection group compared to the control group (Figures 2C, D; Supplementary Table 4). In addition, *Ilumatobacter, Rhodococcus, Halioglobus*, and *Pediococcus* increased significantly in the infected *A. japonicus* (Figures 2E, F). These variations in microbial abundance were synergistic with the tight junction protein expression. Therefore, the alteration of mid-intestine permeability was closely related to the midgut microbiome.

### 3.3. SCFA contents in the midgut were altered in response to the *V. splendidus* infection

**Figure 3:**
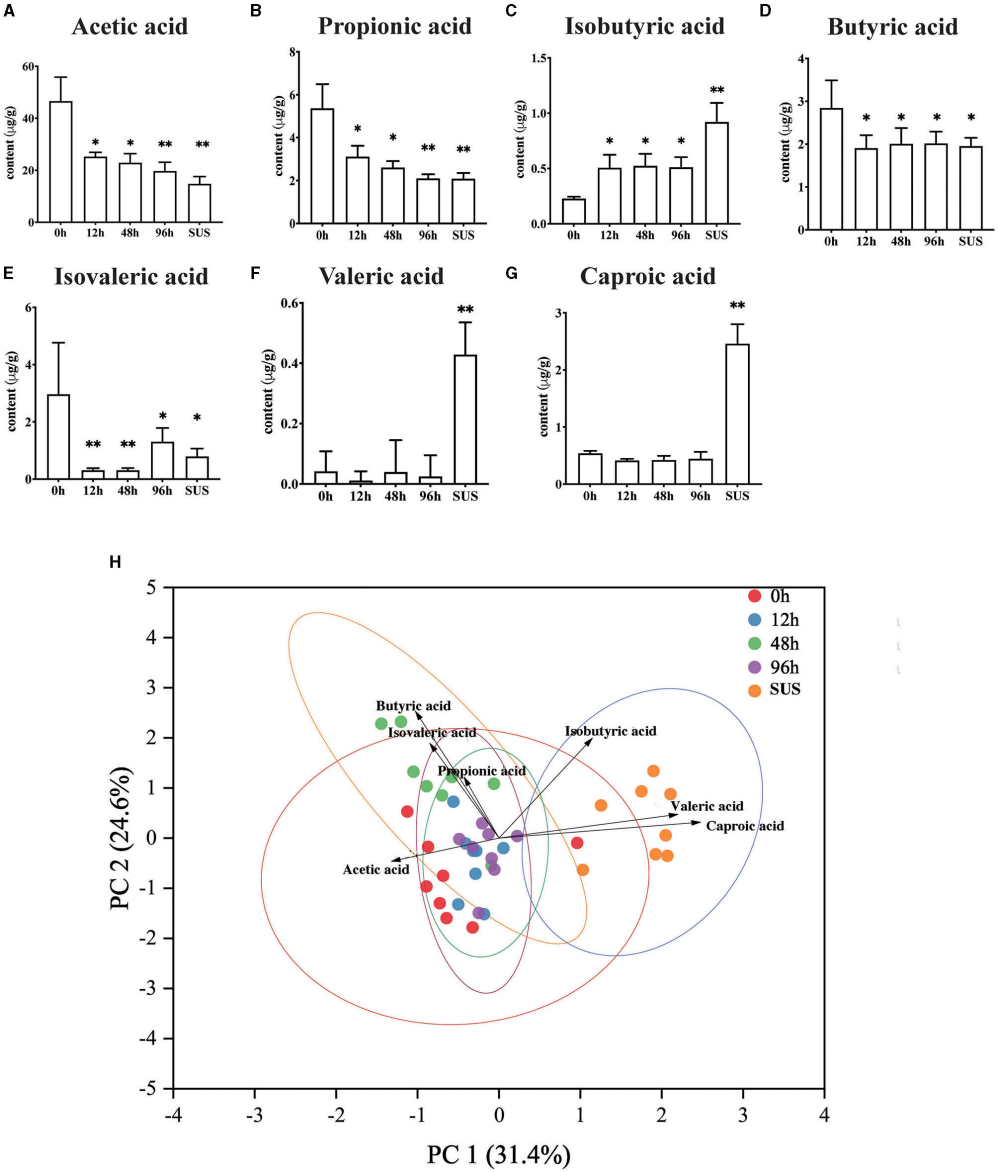
Effect of *V. splendidus* infection on SCFAs in the *A. japonicus* mid-intestine. **(A)** Acetic acid, **(B)** Propionic acid, **(C)** Isobutyric acid, **(D)** Butyric acid, **(E)** Isovaleric acid, **(F)** Valeric acid, and **(G)** Caproic acid. **(H)** Principal component analysis of targeted SCFAs. Asterisks indicate significant differences: *p < 0.05 and **p < 0.01.

The result suggested that there were varying degrees of changes in the SCFA contents in the midgut. Acetic acid gradually decreased and reached the lowest level in the SUS sea cucumber, at 14.84 μg/g (Figure 3A). The propionic acid content in the 12 and 48 h groups was significantly lower than that in the 0 h group (*p* < 0.05), and it decreased to the lowest level in the 96 h and SUS groups, at 2.10 and 2.09 μg/g, respectively (Figure 3B). The butyric acid content of the infection groups was remarkably lower than that of the 0 h group (*p* < 0.05), but there was no remarkable difference among the four infection groups (Figure 3D). The isobutyric acid content in the 12, 48, and 96 h groups was significantly higher than that in the 0 h group (*p* < 0.05), and it increased to the highest level in the SUS group, at 0.71 μg/g (Figure 3C). The isovaleric acid content decreased afterward to the lowest level at 12 and 48 h, both at 0.31 μg/g, and it increased to a slightly higher level at 96 h and in the SUS group but remained remarkably lower than in healthy *A. japonicus* (Figure 3E). There were no significant differences in valeric acid and caproic acid levels associated with pathogen infection, but they showed a substantial increase in SUS-diseased *A. japonicus* (Figure 3F). Therefore, acetic acid had the highest content and the most regular variation among the seven SCFAs.

PCA analysis revealed that the 0 h samples were distinct from those of the SUS group, using the quantification of seven SCFAs as variables and two principal component scores of PC1 and PC2 (31.4 and 24.6% of the explained variance, respectively; Figure 3H). The PCA plot showed a clear separation in the midgut SCFAs between the healthy and SUS sea cucumbers; it also showed that acetic acid was significantly correlated with healthy populations, and valeric and caproic acids were correlated with SUS populations. Then, we employed the OPLS-DA model on SCFAs and observed that the VIP values of acetic acid, valeric acid, caproic acid, and isobutyric acid were >1; among them, the acetic acid VIP value of 1.14 was the highest, as shown in Supplementary Table 5.

### 3.4. Corelationship analysis between the midgut microbiota and SCFAs

**Figure 4:**
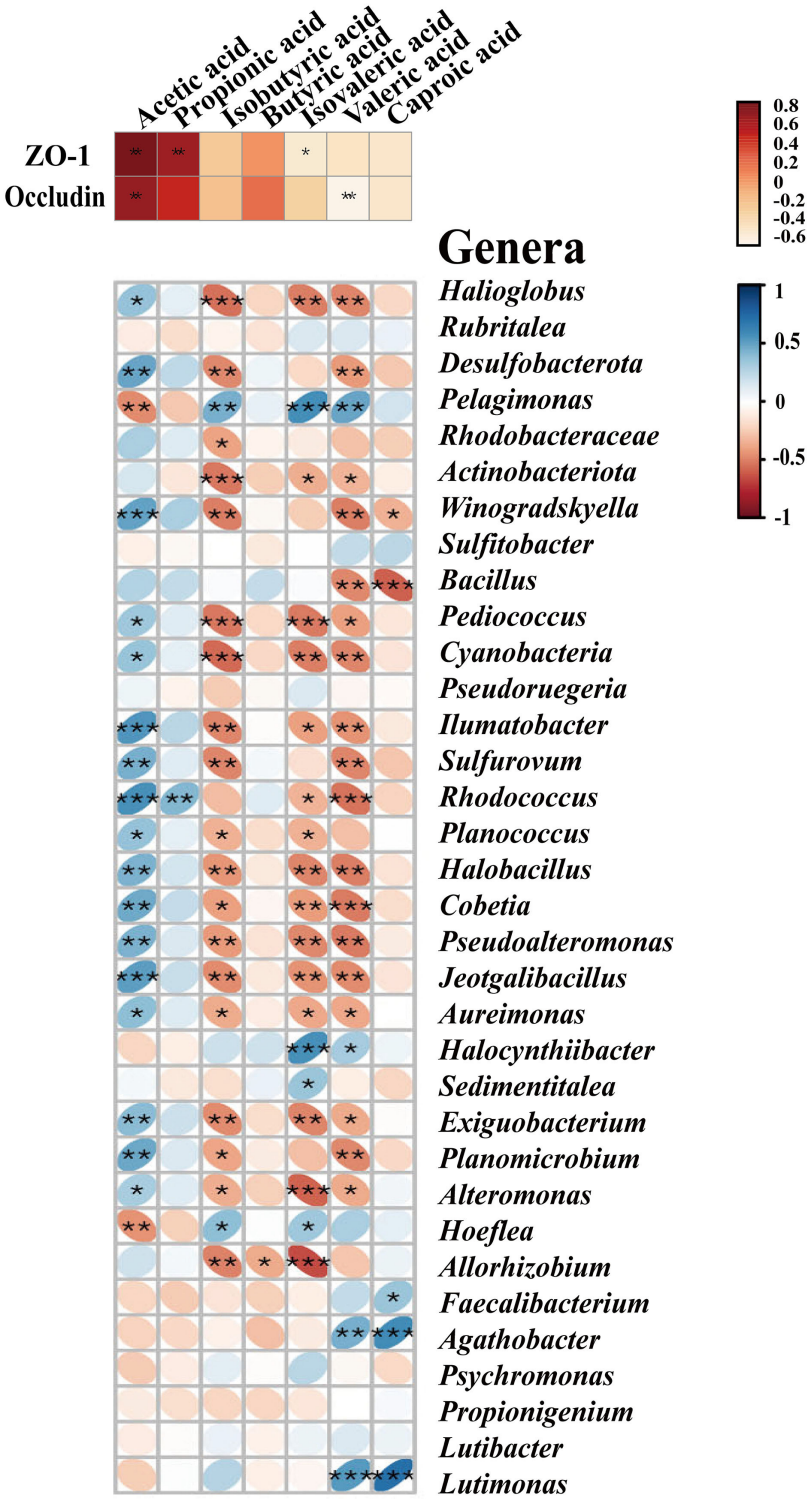
SCFAs exhibit correlations with tight junction proteins and the major midgut microbiota by Spearman's correlation analysis. The color and thickness of the ellipse represent the correlation coefficient value. Asterisks indicate obvious correlations. Asterisks indicate significant differences: *p < 0.05, **p < 0.01, and **p < 0.001.

To explore which resident taxa interact with the SCFAs, we performed the conjoint analysis in R software to investigate the correlation between the midgut microbiota and seven SCFAs. We observed 34 major genera of bacteria associated with the seven SCFAs, of which 17 genera were significantly positively correlated with acetic acid, including *Winogradskyella, Alteromonas, Halioglobus, Bacillus, Pediococcus, Ilumatobacter*, and *Desulfobacterota* (*p* < 0.05), and only *Hoeflea* and *Pelagimons* were significantly negatively correlated (*p* < 0.01) (Figure 4). *Rhodococcus* had a significant correlation with propionic acid, and butyric acid had an obvious negative correlation with only *Allorhizobium* (*p* < 0.05). Similar to the bacteria that showed a significant negative correlation with isobutyric acid, isovaleric acid (*p* < 0.05), and valeric acid, most other bacteria were positively correlated with acetic acid, including *Winogradskyell, Desulfobacterota, Rhodococcus*, and *Sulfurovum*. Both valeric acid and caproic acid were positively correlated with *Agathobacter* and *Lutimonas*. Additionally, valeric acid had a significant positive correlation with *Pelagimonas*, and caproic acid had a significant positive correlation with *Faecalibacterium* (*p* < 0.05) (Supplementary Table 6).

### 3.5. Crosstalk analysis between SCFAs and the tight junction

To further investigate the relationship between SCFAs and intestinal permeability, we analyzed the crosstalk data and compared them between the midgut SCFAs and tight junction proteins based on Spearman's correlation coefficients. As shown in Figure 4 and Supplementary Table 7, there was a strong correlation between the protein levels of SCFAs and the tight junction. Acetic acid was positively correlated to the level of ZO-1 (*r* = 0.93548, *p* < 0.05) and occludin (*r* = 0.89606, *p* < 0.01). Propionic acid (*r* = 0.84230, *p* < 0.01) and isovaleric acid (*r* = −0.54783, *p* < 0.01) were only positively correlated to the level of ZO-1, but there was no significant correlation with occludin. Isovaleric acid was only remarkably negatively correlated with ZO-1 (*r* = −0.41219, *p* < 0.05). Isobutyric acid, butyric acid, and caproic acid had no notable correlation with the tight junction (Figure 4).

### 3.6. Acetate-regulated midgut barrier function

**Figure 5:**
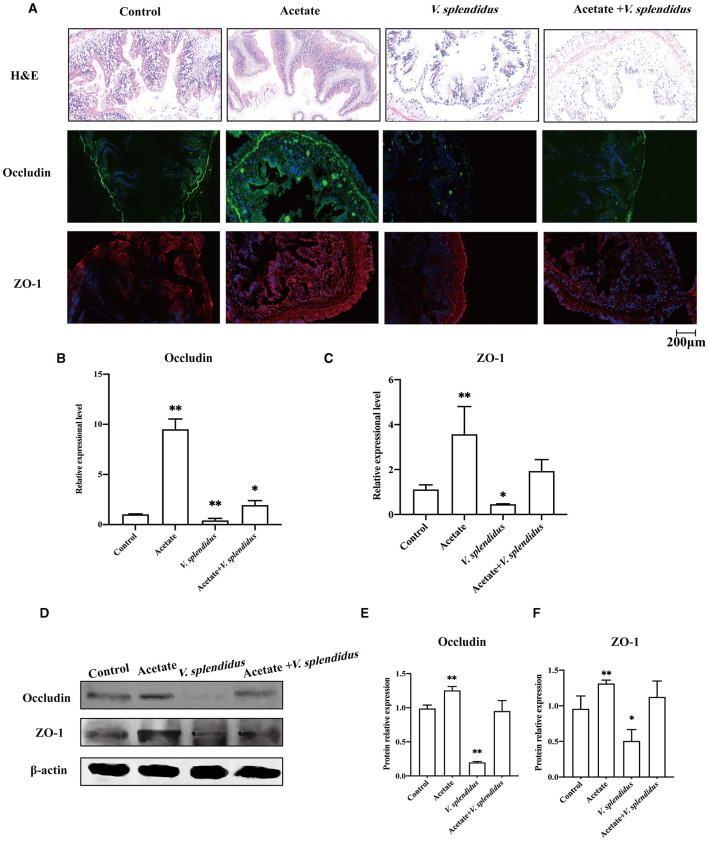
Acetate administration reinforces the integrity of the epithelial barrier during *V. splendidus* infection. **(A)** H&E staining of the section of the *A. japonicus* midgut. **(B)** Immunofluorescence of ZO-1 and occludin in the *A. japonicus* mid-intestine. **(C, D)** RT-PCR analysis of the occludin and ZO-1 transcript levels from the mid-intestine tissue after acetate treatment. **(E, F)** The Western blotting was used to determine the expression level of occludin and ZO-1 with β-actin as the reference in the mid-intestine tissue. Asterisks indicate significant differences: *p < 0.05 and **p < 0.01.

We further studied the regulatory effect of acetate on the intestinal barrier integrity in the midgut by adding acetate to the *A. japonicus* normal feed. The results showed that acetate alleviated the damage caused by *V. splendidus* to the midgut barrier function (Figure 5A). The immunofluorescence result showed that the occludin and ZO-1 fluorescence signals aggravatingly increased compared with the control group (Figure 5A) and almost disappeared in *V. splendidus*. We further measured the mRNA level of occludin and ZO-1 in the midgut tissue by qRT-PCR after acetate treatment; the mRNA significantly increased to 9.22- and 3.57-fold, respectively, in the acetate group compared with the control group (*p* < 0.01; *p* < 0.01). *Vibrio splendidus* significantly reduced the mRNA level, and acetate restored the tight junction mRNA level to the control level (Figures 5B, C). The Western blotting results suggested that acetate restored the occludin and ZO-1 expression levels of the control group in the presence of *V. splendidus* (Figures 5D–F).

### 3.7. Acetate-altered F-actin and ZO-1 localization and organization

**Figure 6:**
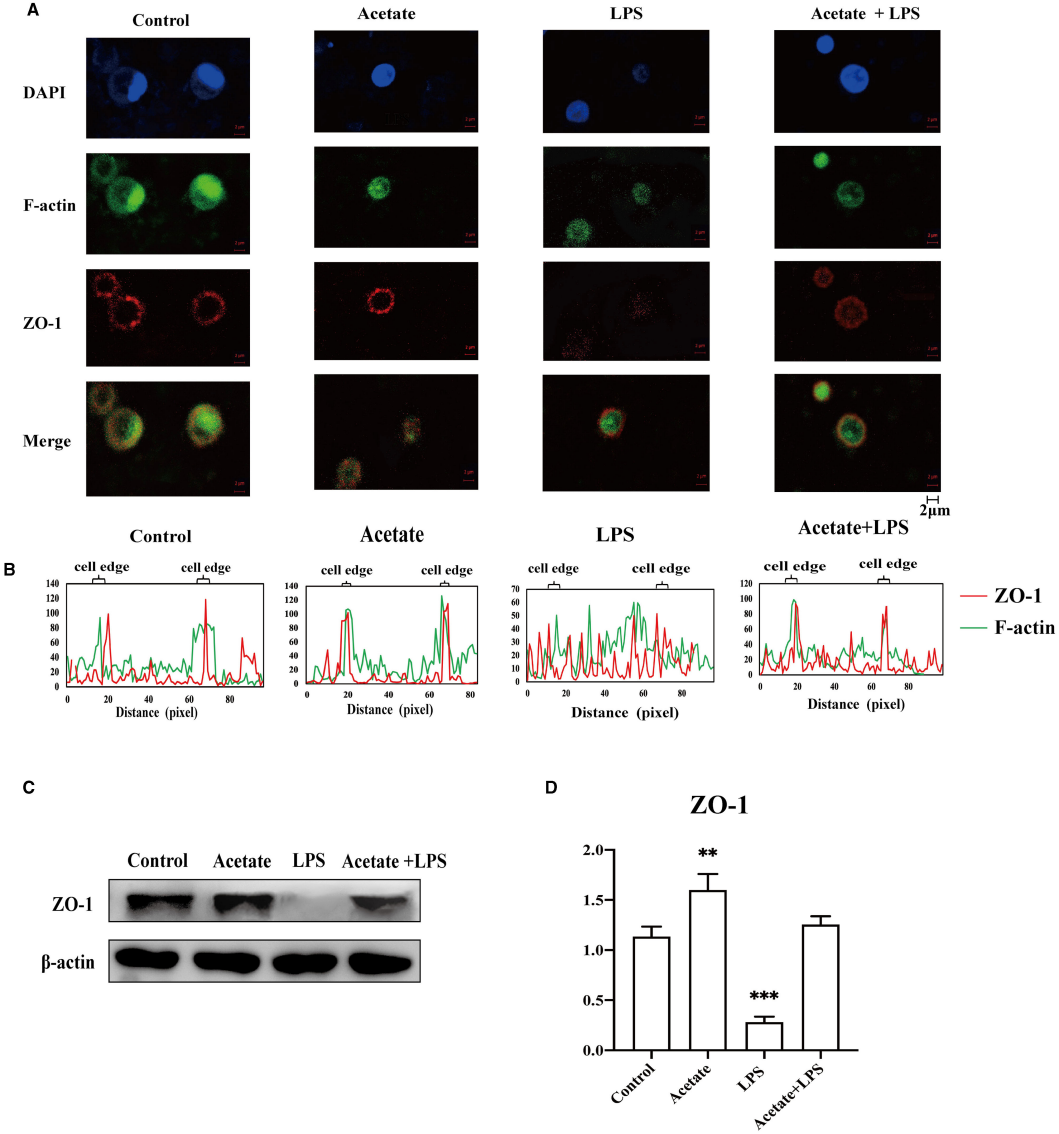
Acetate prevented LPS-induced F-actin and ZO-1 dissociation and reorganization in mid-intestinal epithelial cells. **(A)** Immunofluorescence of ZO-1 and F-actin in the intestinal epithelial cells. **(B)** Fluorescence colocalization analysis for F-actin and ZO-1 of the intestinal epithelial cells analysis. **(C, D)** The Western blotting was used to determine the ZO-1 in the intestinal epithelial cells. Asterisks indicate significant differences: **p < 0.01 and ***p < 0.001.

It was well-documented that actin participated in modulating the tight junction structural integrity via actin reorganization (Zeng and Chi, 2015). To preliminarily understand the detailed mechanism of acetate-mediated midgut permeability, we evaluated the effects of acetate on ZO-1 distribution and F-actin expression in the midgut epithelial cell by separating the epithelial cells from the midgut tissue and culturing *in vitro*. We only evaluated the tight junction ZO-1 expression in our *in vitro* experimental study. The *A. japonicus* intestinal epithelial cells did not form cell lines, and occludin was located at the cell junction. Immunofluorescence analysis revealed that ZO-1 and F-actin in the intestinal epithelial cells showed that acetate reinforces the intestine barrier integrity (Figure 6A). Fluorescence images of the control group showed that ZO-1 was located on the cell border and colocalized with F-actin in the mid-intestinal epithelial cells. In contrast, the cells treated with LPS suggested that ZO-1 and F-actin were weakly expressed in the cell edge and diffused to the cytoplasm (Figures 6A, B). The mid-intestinal epithelial cells incubated with acetic acid showed F-actin and ZO-1 colocalization, and the fluorescence signal at the cell edge was strong (Figure 6B). Compared with the acetate treatment group, the mid-intestinal epithelial cell fluorescence signal of the acetate and LPS treatment group was weak. The Western blotting results suggested that the effect of acetic acid increased the ZO-1 expression in the mid-intestinal epithelial cells (Figures 6C, D). Compared with the LPS-treated mid-intestinal epithelial cells, the combination of acetic acid and LPS could increase the expression of the ZO-1 protein, but it was still lower than that of the control group. Therefore, acetic acid affects the mid-intestinal permeability of *A. japonicus* by affecting F-actin rearrangement and decreasing TJ protein expression.

## 4. Discussion

The most vital function of the intestinal epithelium is being a barrier to prevent intestinal bacteria from entering the blood circulation from the intestinal cavity. The tight junction is regarded as the side gate between the two adjacent cells because it limits the access of molecules according to charges and sizes; it can also regulate paracellular permeability (Bhat et al., 2019). Moreover, the tight junction also plays a palisade function and maintains the polarization of epithelial cells by protecting them from the disorder displacement of apical and basolateral membrane proteins in mammals (Bhat et al., 2019; Heinemann and Schuetz, 2019; Otani and Furuse, 2020). More and more pieces of evidence suggest that configuration and expression changes of TJ proteins are caused by all kinds of stimuli, including toxins (Patterson et al., 2020), ROS (Kim et al., 2019), and nitric oxide (Logsdon et al., 2018). Alterations of the TJ protein level and location are related to the increase in the permeability of the epithelium monolayer bypass (Schilpp et al., 2021). ZO-1 is a cytosolic scaffolding protein, and it is the key molecule in TJ complexes (Lynn et al., 2020). It interacts with other member tight junction compounds (Schwayer et al., 2019) and forms a tight junction complex with the cytoskeleton. In our present study, the mid-intestine barrier integrity could be observed by H&E; the result indicated that *V. splendidus* seriously damaged the mid-intestinal epithelial tissue. The occludin and ZO-1 expression levels were decreased by *V. splendidus* in the midgut tissue, as examined by Western blotting and immunofluorescence (Figure 1). It was demonstrated that the decrease in the occludin and ZO-1 expression levels were involved in the defective barrier morphology and function induced by various pathological factors (Zeng and Chi, 2015). Among pathological factors, cytoskeletal remodeling was caused by the contraction of actin, which destroyed the TJ structure to increase intestinal permeability. The junctional ring of F-actin and myosin II supporting the tight junction is essential for the regulation of physiological and pathophysiological barriers (Madara and Pappenheimer, 1987). For instance, the activation of peripheral myosin light chain kinase (MLCK) is sufficient for enhancing cell bypass permeability (Shen et al., 2006). The activation of MLCK can cause phosphorylation of the myosin light chain (MLC); it can mediate actin contraction and destroy the tight junction, thereby increasing the permeability of epithelial cells. The mechanism by which *V. splendidus* destroys the intestinal barrier and reduces the occludin and ZO-1 expression remains to be further studied. *V. splendidus* might regulate MLCK by activating NF-κB to redistribute tight junction proteins and the surrounding cytoskeletal proteins, resulting in the displacement of the tight junction structure as well as an increase in intestinal permeability. At present, whether tight junction protein downregulation is caused by pathogenic microorganisms or a pathological state is not clear. Some studies indicate that the MAPK signaling pathway can regulate the transport of intestinal epithelial cells by modulating the expression of tight junction proteins and their phosphorylation degree (Basuroy et al., 2006).

In addition to the destruction of the intestinal barrier, we also found that the infection of *V. splendidus* altered the intestinal microbial community structure and microbiota-derived SCFA profiling (Figures 2, 3), The result indicated that microbiota-derived SCFAs may exert a function in SUS *A. japonicus*. *Vibrio splendens* invasion could influence the intestinal microbiota homeostasis by decreasing the alpha diversity of the intestinal microbiota in *A. japonicus* and decreasing the abundance of gram-positive Firmicutes and gram-negative Desulfobacterota as the most abundant phyla in the *A. japonicus* gut (Zhang et al., 2018). The imbalance of the intestinal microbiota may lead to the proliferation of previously conditioned pathogens in the gut (Mallon et al., 2015). The decrease in the bacterial community diversity may lead to a decrease in the functional stability of bacterial communities, resulting in an increased disease risk (Xiong et al., 2019). Ecological evidence suggests that a decrease in gut bacterial diversity can provide a vacancy for microbial invaders (Ruben et al., 2005). Recent studies have revealed that many illnesses are always accompanied by the alteration of the intestinal microbiota aberrations and SCFA profiling (Madara and Pappenheimer, 1987). In this study, the characterization of the intestinal bacteria of *A. japonicus* revealed a dominance of Proteobacteria, Firmicutes, Bacteroidetes, and Actinobacteriota, which are typical dominant members of the vertebrate gut, particularly in mammals (Turnbaugh et al., 2007; Mahowald et al., 2009). The finding regarding these dominant phyla was similar to that of other studies involved in the gut microbiota of *A. japonicus* (Yang et al., 2015; Kwong et al., 2018; Pagán-Jiménez et al., 2019). At the genus level, *Rhodococcus* was obviously decreased during the *V. splendidus* infection, which was representative of the Actinobacterio phylum and can degrade harmful organic substances. Meanwhile, the decrease also occurred in Rhodobacteraceae, which is an aquatic photosynthetic probiotic for the intestinal epithelial cells of tilapias (Zhou et al., 2020). The decrease in Rhodobacteraceae and *Rhodococcus* during the *V. splendidus* infection suggested that *V. splendidus* destroyed the midgut microbiota homeostasis by reducing the abundance of these probiotics. Additionally, the correlation analysis revealed that the bacteria with a significant negative correlation with valeric acid were positively correlated with acetic acid. Many reports have demonstrated that Proteobacteria and Firmicutes are the dominant bacteria producing acetic acid and butyric acid (Kwong et al., 2018), but the variation trend in Proteobacteria and Firmicutes abundances was completely different from that of the valeric acid content. Hence, we speculated that valeric acid and acetic acid might exert a different function in the intestine via the midgut microbiota alteration.

Emerging pieces of evidence suggest that acetic acid and butyric acid derived from the microbiota can influence the host's immune response (Denning et al., 2000; Moore et al., 2001). Specifically, it has been demonstrated that butyric acid maintains mucosal integrity and has an anti-inflammatory effect on the intestine due to its significant antineoplastic properties (Bagheri et al., 2018). Many reports have demonstrated that butyric acid plays a beneficial role in the epithelial barrier formation in humans with IBD or IBS (Sokol et al., 2009; Eeckhaut et al., 2013; Machiels et al., 2014). Zeng and Chi (2015) demonstrated the ability of microbial-derived butyrate to promote the epithelial barrier function through claudin-2 repression in human epithelial cells. However, our results found that propionic acid and butyric acid do not play any role in maintaining the intestinal barrier homeostasis, and they were not identified as marker metabolites in the SCFA profile analysis results (Figure 4). In particular, butyric acid had only one phyla bacterium with a significant correlation. We found that acetic acid had significant differences with the *V. splendidus* infection. There were differences in the living environment and food composition between higher animals and our research subjects. The differences led to changes in the midgut microbiota community and functions and then altered the metabolites profile. Hence, we considered that the synergy between acetic acid and the intestinal permeability variation in the *A. japonicus* midgut is not accidental, but the inevitable result was induced by *V. splendidus* infection in the sea cucumbers. Hu et al. (2023) reported that *Lactobacillus reuteri* abundance significantly decreased in the HCC mice intestine, accompanied by the reduction of SCFA levels, especially acetate.

The mid-intestine tissue morphology alteration and microbiota-derived SCFA profiling indicate that we should seek to understand the correlation between SCFAs and the intestinal epithelial barrier. The result of the association analysis between SCFAs and the tight junction confirmed that there was a strong correlation between SCFAs and the intestinal barrier (Figure 4), but only acetic acid was significantly correlated with both occludin and ZO-1. Meanwhile, microbial aberration and SCFA contents also indicate that acetic acid plays an essential role in SUS occurrence. To test the function of acetic acid on the intestinal barrier, we added acetate to the normal forage to feed the sea cucumbers. Our study found that acetate could maintain the normal mid-intestine barrier integrity by increasing the occludin and ZO-1 expression under *V. splendidus* invasion (Figure 5). The result was consistent with those of other studies, which demonstrated that SCFAs secure the intestinal barrier function by regulating the tight junction expression (Yang et al., 2010; Arpaia et al., 2013). These data indicate that *V. splendidus* reduced the expression levels of occludin and ZO-1, whereas acetate successfully prevented the *V. splendidus*-induced effects. However, the microbiota-derived SCFAs' mechanism that led to the improvement of the intestinal barrier and protected the intestinal barrier from exogenous bacteria is unknown. To further confirm the association between acetic acid and the mid-intestine barrier function regulated by the tight junction expression and distribution, acetate was employed to treat mid-intestinal epithelial cells. This study found that the ZO-1 and F-actin disaggregation at the cell border and F-actin recombination occurred in the LPS-treated cells, and acetate could maintain the normal distribution of F-actin and ZO-1 to cope with the challenge posed by LPS (Figure 6). Peerapen and Thongboonkerd (2021) also indicate that various stimuli induce F-actin reorganization. Cytoskeleton, composed of F-actin and other partner proteins, plays a key role in the modulation and formation of the cell shape (Schakenraad et al., 2020) and locomotion (Svitkina, 2018). The reorganization of F-actin has been reported to influence the tight junction structure integrity due to the direct connection of ZO-1 with F-actin (Odenwald et al., 2017; Otani and Furuse, 2020). F-actin contractile is driven by MLCK/MLC pathway activation, which causes tight junction contraction and structural displacement, leading to increased permeability (Hecht et al., 1996; Yang et al., 2010). Therefore, acetate might protect the F-actin organization and the tight junction structure from LPS-induced damage by inhibiting the nuclear import of NF-κB p65 and activating the MLCK/MLC pathway.

## 5. Conclusion

Overall, our results provide evidence that the alteration of the midgut microbiota and SCFA profiling is accompanied by intestinal barrier damage and tight junction downregulation during *V. splendidus* infection in sea cucumbers. Moreover, the SCFA profiling analysis suggested that acetic acid was a characteristic metabolite in sea cucumbers. The feeding experiment also confirmed that acetic acid has a profound effect on the intestinal barrier integrity and tight junction structure integrity.

## Data availability statement

The datasets presented in this study can be found in online repositories. The names of the repository/repositories and accession number(s) can be found in the article/Supplementary material.

## Ethics statement

The animal study was approved by Experimental Animal Ethics Committee of Ningbo University, China. The study was conducted in accordance with the local legislation and institutional requirements.

## Author contributions

CL: Conceptualization, Funding acquisition, Project administration, Supervision, Writing—review and editing. MS: Investigation, Methodology, Validation, Writing—original draft. ZZ: Funding acquisition, Supervision, Validation, Writing—review and editing. YL: Formal analysis, Validation, Writing—review and editing. YX: Supervision, Writing—review and editing.
