# Supplementary material for: Midgut microbiota affects the intestinal barrier by producing short-chain fatty acids in Apostichopus japonicus
Source: Front Microbiol. 2023 Oct 17;14:1263731. doi: 10.3389/fmicb.2023.1263731 (PMC10616862; doi:10.3389/fmicb.2023.1263731)
Supplement: Supplementary file 1 [file Table_1.DOCX]

**Supplement materials**

TABLE S1 Linear regression equation, precision, repeatability and limit of quantitation of seven SCFAs standards

| SCFA | Retention time (min) | | | Quantitative ion | | | linear equation | Correlation coefficient (r) | | Linear range (μg/ml) |
| --- | --- | --- | --- | --- | --- | --- | --- | --- | --- | --- |
| Acetic acid | | 4.21 | | | 60 | | y =0.0227x ± 0.0108 | | 0.9907 | 0.02-500 |
| Propionic acid | | 5.21 | | | 74 | | y=0.0236x ± 0 | | 0.9968 | 0.02-500 |
| Isobutyric acid | | | 5.6 | | 73 | | y=0.0295x ± 4e-04 | | 0.9985 | 0.02-500 |
| Butyric acid | | | 6.44 | | 60 | | y=0.0713x ± 6e-04 | | 0.999 | 0.02-500 |
| Isovaleric acid | | | 7.08 | | 60 | | y=0.0805x ± 5e-04 | | 0.9989 | 0.02-500 |
| Valeric acid | | | 8.17 | | 60 | | y=0.0822x ± 2e-04 | | 0.99874 | 0.02-500 |
| Capric acid | | | 9.82 | | | 60 | y=0.074x ± 0.0016 | | 0.9979 | 0.02-500 |

TABLE S2 Primer used in this study

| Primer | Sequence (5’-3’) | Application |
| --- | --- | --- |
| ZO-1 | F: CTGGTGTCAGCATCAAGATTCC  R: CAGTATGGGGCAGGTTGAGTTC | Real-time PCR |
| Occludin | F: CACAGAATTTCCACCAATCACAAC  R: GTACTCATCGTCTTGAAGTCTGCTC | Real-time PCR |
| $\beta$-actin | F: CCATTCAACCCTAAAGCCAACA  R: ACACACCGTCTCCTGAGTCCAT | Real-time PCR |

TABLE S3 Bacterial taxonomic profiling at the phylum level in sea cucumber. Values presented here are average$\pm$SD

| Phylum | 0 h | 12 h | 48 h | 96 h |
| --- | --- | --- | --- | --- |
| Proteobacteria | 43.45±6.65 | 28.20±4.73 | 29.75±12.33 | 66.32±8.81 |
| Firmicutes | 22.86±8.77 | 49.43±9.38 | 35.41±14.53 | 16.90±8.11 |
| Bacteroidota | 5.41±2.93 | 6.84±7.80 | 5.39±3.52 | 3.45±2.99 |
| Actinobacteriota | 12.03±2.83 | 7.26±2.69 | 4.81±2.26 | 5.68±2.28 |
| Verrucomicrobiota | 2.25±1.34 | 1.04±0.39 | 1.36±0.81 | 2.56±1.59 |
| Cyanobacteria | 2.61±0.83 | 1.65±0.88 | 0.84±0.43 | 1.14±0.70 |
| Desulfobacterota | 5.69±2.94 | 2.69±0.78 | 2.06±0.83 | 1.85±0.95 |
| Campilobacterota | 2.06±1.99 | 0.61±0.54 | 0.79±0.50 | 0.32±0.14 |
| Fusobacteriota | 0.38±0.53 | 0.12±0.09 | 0.22±0.15 | 0.29±0.47 |
| Chloroflexi | 0.81±0.28 | 0.30±0.19 | 0.25±0.14 | 0.47±0.45 |
| Patescibacteria | 0.37±0.01 | 0.17±0.12 | 0.13±0.00 | 0.08±0 |
| Acidobacteriota | 0.47±0.28 | 0.36±0.27 | 0.14±0.00 | 0.22±0.06 |
| Myxococcota | 0.28±0.08 | 0.11±0.05 | 0.09±0.00 | 0.07±0.04 |
| Nitrospirota | 0.09±0.06 | 0.12±0.11 | 0.03±0.00 | 0.12±0.11 |
| Others | 1.23±0.52 | 1.1±0.83 | 0.44±0.24 | 0.53±0.08 |

TABLE S4 Midgut bacterial taxonomic profiling at the family level in sea cucumber. Values presented here are average$\pm$SD

| Family | 0 h | 12 h | 48 h | 96 h |
| --- | --- | --- | --- | --- |
| Rhodobacteraceae | 13.81±8.46 | 10.06±3.4 | 23.51±3.25 | 53.43±9.91 |
| Bacillaceae | 6.73±2.89 | 37.76±16.4 | 36.44±7.07 | 4.51±1.22 |
| Flavobacteriaceae | 1.45±1.63 | 1.19±1.5 | 2.11±2.29 | 0.39±0.53 |
| Rhizobiaceae | 2.37±1.08 | 2.38±1.14 | 2.9±2.76 | 4.19±3.02 |
| Nocardiaceae | 2.81±1.51 | 2.29±1.3 | 1.92±0.97 | 0.86±0.67 |
| Psychromonadaceae | 0.07±0.05 | 0.12±0.08 | 0.46±0.61 | 0.13±0.11 |
| Rubritaleaceae | 1.79±1.77 | 0.78±0.34 | 1.52±0.81 | 2.38±1.5 |
| Desulfocapsaceae | 3.91±2.93 | 1.38±0.73 | 1.79±0.38 | 0.6±0.24 |
| Halieaceae | 5.47±5.25 | 1.88±2.46 | 0.91±0.94 | 0.32±0.3 |
| Vibrionaceae | 4.45±2.76 | 3.49±1.65 | 2.03±1.27 | 1.53±1.11 |
| Planococcaceae | 4.02±2.3 | 3.15±1.97 | 1.49±0.43 | 2.16±0.95 |
| Ilumatobacteraceae | 1.78±1.02 | 0.61±0.25 | 0.55±0.14 | 0.28±0.07 |
| Halomonadaceae | 3.03±2.71 | 1.43±0.94 | 0.65±0.32 | 0.65±0.52 |
| Pseudoalteromonadace | 1.95±1.48 | 1.05±0.6 | 0.39±0.19 | 0.54±0.52 |
| Enterobacteriaceae | 0.71±0.33 | 0.58±0.21 | 0.55±0.23 | 0.58±0.35 |
| Sulfurovaceae | 2.03±1.86 | 0.54±0.46 | 0.84±0.65 | 0.3±0.14 |
| Alteromonadaceae | 1.3±1.02 | 0.65±0.39 | 0.24±0.17 | 0.09±0.05 |
| Colwelliaceae | 0.17±0.08 | 0.22±0.12 | 0.44±0.21 | 0.25±0.14 |
| Exiguobacteraceae | 36.58±11.51 | 26.62±15.09 | 18.89±6.18 | 22.97±11.19 |
| Others | 38.01±11.50 | 31.09±5.15 | 12.99±3.46 | 18.17±6.92 |

TABLE S5 OPLS-DA model analysis of short chain fatty acid for VIP value

| Var ID | VIP |
| --- | --- |
| Acetic acid | 1.138 |
| Valeric acid | 1.131 |
| Caproic acid | 1.129 |
| Isobutyric acid | 1.048 |
| Isovaleric acid | 0.980 |
| Propionic acid | 0.763 |
| Butyric acid | 0.713 |

TABLE S6 Spearman’s correlation coefficient matrix between midgut microbiota and SCFAs with *V. splendidus* infection

| Genera | Acetic acid | Propionic acid | Isobutyric acid | Butyric acid | Isovaleric acid | Valeric acid | Caproic acid |
| --- | --- | --- | --- | --- | --- | --- | --- |
| Halioglobus | 0.376 | 0.101 | -0.534 | -0.202 | -0.499 | -0.474 | -0.191 |
| Rubritalea | -0.092 | -0.171 | -0.058 | -0.142 | 0.157 | 0.159 | 0.086 |
| Desulfobacterota | 0.495 | 0.242 | -0.465 | 0.068 | -0.180 | -0.417 | -0.253 |
| Pelagimonas | -0.457 | -0.260 | 0.459 | 0.099 | 0.588 | 0.496 | 0.182 |
| Rhodobacteraceae | 0.307 | 0.139 | -0.380 | -0.059 | -0.102 | -0.279 | -0.233 |
| Actinobacteriota | 0.168 | -0.128 | -0.513 | -0.237 | -0.349 | -0.327 | -0.082 |
| Winogradskyella | 0.506 | 0.304 | -0.497 | -0.032 | -0.238 | -0.496 | -0.332 |
| Sulfitobacter | -0.072 | -0.030 | -0.006 | -0.111 | 0.004 | 0.232 | 0.256 |
| Bacillus | 0.283 | 0.237 | 0.027 | 0.224 | 0.029 | -0.466 | -0.580 |
| Pediococcus | 0.344 | 0.126 | -0.515 | -0.188 | -0.504 | -0.400 | -0.129 |
| Cyanobacteria | 0.362 | 0.104 | -0.552 | -0.192 | -0.490 | -0.479 | -0.135 |
| Pseudoruegeria | 0.063 | -0.055 | -0.246 | -0.034 | 0.146 | -0.046 | -0.026 |
| Ilumatobacter | 0.553 | 0.264 | -0.470 | -0.016 | -0.398 | -0.432 | -0.118 |
| Sulfurovum | 0.456 | 0.129 | -0.476 | 0.050 | -0.156 | -0.477 | -0.266 |
| Rhodococcus | 0.574 | 0.424 | -0.299 | 0.120 | -0.325 | -0.526 | -0.218 |
| Planococcus | 0.376 | 0.105 | -0.339 | -0.175 | -0.340 | -0.288 | 0.005 |
| Halobacillus | 0.450 | 0.174 | -0.427 | -0.119 | -0.478 | -0.491 | -0.148 |
| Cobetia | 0.475 | 0.221 | -0.390 | -0.044 | -0.409 | -0.507 | -0.174 |
| Pseudoalteromonas | 0.450 | 0.153 | -0.411 | -0.148 | -0.466 | -0.501 | -0.110 |
| Jeotgalibacillus | 0.521 | 0.217 | -0.453 | -0.118 | -0.436 | -0.458 | -0.140 |
| Aureimonas | 0.399 | 0.138 | -0.350 | -0.099 | -0.368 | -0.366 | -0.009 |
| Halocynthiibacter | -0.206 | -0.085 | 0.208 | 0.194 | 0.580 | 0.323 | 0.061 |
| Sedimentitalea | 0.049 | -0.112 | -0.161 | 0.086 | 0.357 | -0.083 | -0.205 |
| Exiguobacterium | 0.408 | 0.202 | -0.458 | -0.173 | -0.477 | -0.351 | -0.020 |
| Planomicrobium | 0.482 | 0.140 | -0.373 | -0.103 | -0.284 | -0.465 | -0.198 |
| Alteromonas | 0.316 | 0.125 | -0.345 | -0.229 | -0.577 | -0.353 | 0.050 |
| Hoeflea | -0.435 | -0.226 | 0.400 | 0.011 | 0.346 | 0.307 | 0.101 |
| Allorhizobium | 0.198 | 0.044 | -0.488 | -0.356 | -0.648 | -0.267 | 0.073 |
| Faecalibacterium | -0.204 | -0.249 | -0.139 | -0.228 | -0.088 | 0.235 | 0.357 |
| Agathobacter | -0.214 | -0.196 | -0.067 | -0.283 | -0.103 | 0.454 | 0.599 |
| Psychromonas | -0.241 | -0.099 | 0.113 | -0.020 | 0.267 | -0.037 | -0.189 |
| Propionigenium | -0.104 | -0.168 | -0.201 | -0.200 | -0.129 | -0.004 | 0.038 |
| Lutibacter | -0.100 | -0.012 | 0.086 | -0.063 | 0.079 | 0.141 | 0.076 |
| Lutimonas | -0.233 | 0.014 | 0.277 | -0.080 | -0.042 | 0.536 | 0.724 |

TABLE S7 Spearman’s correlation coefficient matrix between SCFAs and tight junction with *V. splendidus* infection

| SCFAs | Occludin | ZO-1 |
| --- | --- | --- |
| Acetic acid | 0.935 | 0.896 |
| Propionic acid | 0.479 | 0.842 |
| Isobutyric acid | -0.479 | -0.321 |
| Butyric acid | 0.090 | 0.194 |
| Isovaleric acid | -0.534 | -0.412 |
| Valeric acid | -0.548 | -0.499 |
| Caproic acid | -0.328 | -0.420 |
